# Unusual cardiovascular complications of brucellosis presenting in two men: two case reports and a review of the literature

**DOI:** 10.1186/1752-1947-5-22

**Published:** 2011-01-20

**Authors:** Nikolaos K Gatselis, Konstantinos P Makaritsis, Ioannis Gabranis, Aggelos Stefos, Konstantinos Karanikas, George N Dalekos

**Affiliations:** 1 Department of Medicine, Medical School, University of Thessaly, Larissa, Greece; 2 Second Department of Medicine, General Hospital of Larissa, Greece

## Abstract

**Introduction:**

Brucellosis is a zoonosis with worldwide distribution, which is particularly endemic in many countries of the Mediterranean basin. Cardiovascular complications of this disease, such as endocarditis, myocarditis and pericarditis, are very rare, with even fewer cases of myocarditis or asymptomatic pericardial effusion in the absence of concomitant endocarditis being reported.

**Case presentation:**

We report two cases of brucellosis in two Caucasian men, aged 17 and 34 years old, with myocarditis and asymptomatic pericardial effusion, respectively. Of note, neither patient had concomitant endocarditis. The disease was confirmed serologically and by blood cultures. Both patients recovered completely after receiving appropriate antibiotic treatment without any sign of relapse during a follow-up of 12 months.

**Conclusion:**

These two cases emphasize that in endemic areas *Brucella *can be considered as a potentially causative agent of idiopathic pericardial effusion or myocarditis, even in the absence of concomitant endocarditis. This possibility could be taken into account particularly in cases where contraction of brucellosis is possible, such as occupational exposure or consumption of unpasteurized dairy products.

## Introduction

Brucellosis is a worldwide zoonosis, with the Mediterranean basin, the Middle East, India, Mexico, and Central and South America being the most affected areas [1]. The disease has generalized and systemic symptoms, and almost every organ of the human body can be affected by *Brucella*. Despite this, the incidence of cardiovascular complications in brucellosis, such as endocarditis, myocarditis or pericarditis, is reported to be as low of 1% of cases [1,2], with even fewer cases of myocarditis or pericarditis in the absence of concomitant endocarditis being reported. Indeed, myocarditis, pericarditis or asymptomatic pericardial effusion in brucellosis is thought to develop almost exclusively in the presence of endocarditis [1,2]. We report two cases of patients, one with asymptomatic pericardial effusion and the other myocarditis caused by brucellosis in the absence of concomitant endocarditis. These unusual features of brucellosis may be underestimated components of the disease.

## Case presentations

### Patient 1

A 34-year-old Caucasian man was admitted our hospital with malaise, fatigue, and low-grade fever (up to 38°C), and a two-month history of rigors. He also reported anorexia and weight loss, night sweats, and generalized arthralgia. His medical history was non-contributory. He worked as a food handler.

On physical examination, the only abnormalities were a systolic murmur (grade II/VI) of the heart and hepatosplenomegaly. Laboratory investigations revealed low haemoglobin 11.6 g/dL (normal range 13.0-18.0 g/dL), and raised aspartate aminotransferase (57 U/L; upper normal limit (UNL) 40 U/L), alaninoaminotransferase (57 U/L; UNL: 40 U/L) and gamma-glutamyl transpeptidase (55 U/L; UNL 37 U/L). Electrocardiography (ECG) was normal with sinus rhythm, but chest radiography revealed slight cardiomegaly. Stool, urine and pharynx cultures, and investigations for Epstein-Barr virus (EBV), cytomegalovirus, enteric cytopathic human orphan (ECHO) virus, coxsackie viruses, herpes simplex virus (HSV), varicella-zoster virus (VSV), adenovirus, *Coxiella burnetti*, *Chlamydia pneumoniae*, *Leptospira *spp. and *Mycoplasma pneumoniae *were negative. However, serum agglutination tests were positive (titre >1:2560), and enzyme-linked immunosorbent assay (SERION ELISA Classic IgG/IgM; Institute Virion/Serion GmbH, Wurzburg, Germany) tests for anti-*Brucella *IgG and IgM antibodies were strongly positive (118 U/ml and 35.5 U/ml; UNL 30 U/ml and 20 U/ml, respectively). In addition, *Brucella spp*. were isolated from consecutive blood cultures (six out of eight cultures positive). Two-dimensional trans-thoracic and trans-oesophageal echocardiography was performed to investigate for the possible presence of *Brucella*-related endocarditis because of our patient's systolic heart murmur, six positive blood cultures and presence of cardiomegaly on chest radiographs. The investigation showed normal valves without any sign of vegetation; however, there was marked pericardial effusion without signs of cardiac tamponade, even though our patient had not reported any chest pain.

Additional extensive laboratory blood tests were performed because of the asymptomatic pericardial effusion, including tumor markers, C-reactive protein, fibrinogen, rheumatoid factor, antinuclear antibodies, anti-double-stranded DNA antibodies, serum immunoglobulins, and C3 and C4 component, but did not reveal the cause. There were no clinical or radiologic signs (computed tomography and MRI scans) suggestive for the presence of spondylitis, splenic disease or epididymoorchitis.

Accordingly, a diagnosis of *Brucella*-related asymptomatic pericardial effusion in the absence of concomitant endocarditis was made and specific treatment with antibiotics only was started immediately. Our patient was given oral doxycycline 100 mg twice daily plus oral rifampicin 900 mg once daily for three months, with intramuscular streptomycin 1 g once daily for the first three weeks. Our patient attended for treatment and follow-up to our outpatient clinic every two weeks. His symptoms regressed rapidly 10 days after initiation of treatment, and no recurrence or adverse drug reactions were observed during follow-up (to one year from the beginning of therapy). Echocardiographic studies three, six and 12 months after the start of treatment showed no pericardial effusion. When last seen, 12 months after starting therapy, our patient was well with no sign of relapse.

### Patient 2

A 17-year-old Caucasian man was admitted to our hospital with a six-day history of high-grade fever (up to 39°C) and chest pain. He also reported fatigue, anorexia, generalized arthralgia, headache and night sweats. He had been treated with paracetamol by his general practitioner for an upper respiratory infection, but the symptoms remained and worsened. He was living in a rural area of central Greece and was working as a stock farmer. His medical history was unremarkable.

On physical examination, only tachypnoea and hepatosplenomegaly were seen. ECG demonstrated repolarization disturbances with T-wave inversion in the II, III, aVF and V2 to V6 leads.

Laboratory investigations found a raised white blood cell count of 11.15 ՠ10^9^/l (normal range 4-11 ՠ10^9^/L), creatine phosphokinase 305 IU/L (30-190 IU/L) and lactate dehydrogenase 247 IU/L (12-230 IU/L). Results of the remaining haematological and biochemical tests, including liver function tests, were within normal ranges. A qualitative determination of troponin was positive, which was confirmed by a quantitative method.

A diagnosis of myocarditis due to an infectious agent was suspected because of the history of chest pain in association with the positive troponin test and the ECG abnormalities. Two-dimensional trans-thoracic and trans-oesophageal echocardiography revealed a mild regional motion abnormality of the left ventricular wall but without pericardial effusion or valves vegetations. Blood, urine, and pharynx cultures were negative, as were serology tests for Influenza A and B, parvovirus, EBV, CMV, ECHO virus, coxsackie virus, HSV, VSV, adenovirus, *Coxiella burnetti*, *Chlamydia pneumonia*, *Leptospira*, *Mycoplasma pneumonia*. However, serum agglutination tests were positive at a titre >1:1280 and ELISA results for anti-Brucella IgG and IgM antibodies were strongly positive (92 U/ml and 48 U/ml, antibodies, respectively), and *Brucella spp *was isolated in two out of four sets of blood cultures. As for patient 1, there were no clinical and radiologic signs suggestive for the presence of other systemic features of the disease.

Accordingly, a diagnosis of *Brucella*-related myocarditis was made and our patient was treated only with the specific triple antibiotic regimen as for patient 1 (oral doxycycline 100 mg twice daily plus oral rifampicin 900 mg once daily for three months, with intramuscular streptomycin 1 g once daily for the first three weeks). His symptoms regressed rapidly after initiation of treatment. His echocardiographic and ECG studies three and 12 months after the start of treatment were completely normal, and he showed no signs of relapse during 12 months of follow-up at our outpatient clinic.

## Discussion

Brucellosis is still highly prevalent in countries of the Mediterranean basin including Greece [1,3-5]. Based on the well-known routes of transmission, brucellosis has long been considered an occupational disease affecting shepherds, abattoir workers, food handlers, veterinarians, dairy-industry professionals, and personnel in microbiologic laboratories [1,4-6]. The clinical features of the disease include splenomegaly, joint involvement presenting as arthritis or spondylodiscitis, lymphadenopathy, lung or liver involvement, and orchitis or epididymoorchitis [1,7-9].

Cardiovascular complications of brucellosis (endocarditis, myocarditis and pericarditis) are rare. In cases of endocarditis, which is the most common cardiovascular involvement of the disease, the aortic valve and less frequently the mitral valve are affected. No good response to medical therapy has been observed, and there is poor prognosis without surgery [10]. Replacement of the affected valve is almost always necessary after five to seven days of antibiotic therapy [10]. In these cases, the development of pericardial effusion or myocarditis is common. However, in the absence of concomitant *Brucella*-related endocarditis, development of pericardial effusion and/or myocarditis seems to be extremely rare [1,2,11-18]. Indeed, when we searched in the MEDLINE database for articles published between 1984 and 2010 using the words 'pericarditis', 'myocarditis' and 'brucellosis', we identified only 11 reports in the English literature of adult patients with brucellosis including 14 patients with appropriate information for analysis on brucellosis-related pericarditis or myocarditis in the absence of concomitant endocarditis [2,12,14,17,19-25] (Table 1). In addition, pericarditis or myocarditis was not reported in any recently reported cases (>200) of brucellosis [1,13]. In another study from Spain (Malaga), it was reported that only 1.5% of 530 brucellosis cases had cardiac involvement, with only one patient with pericarditis and one with myocarditis (0.2%) in the absence of concomitant endocarditis (Table 1) [2]. In a prospective study of 400 cases from Kuwait, only two cases with endocarditis and an additional six cases of other cardiovascular complications (including one case of myocarditis) were reported (Table 1) [19]. Lubani *et al. *[26] reported three more cases of *Brucella *myocarditis in a series of 200 children with brucellosis, and myocarditis has also been described in a case of disseminated brucellosis with multiple organ involvement [20].

**Table 1:** Review of the clinical characteristics, treatment and outcome of the reported adult patients in the English literature with Brucellosis-related pericarditis and/or myocarditis in the absence of concomitant endocarditis.

Publication (Ref)	Cases, *n*	Clinical presentation	ECG	Echocardiography	Serum agglutination test	Cultures	Pericarditis	Myocarditis	Treatment	Outcome
Pandit *et al*., 2010 [21]	1	Fever, dyspnea, constitutional symptoms^a^	Sinus tachycardia	Abnormal	1:1280	Positive	No	Yes	Streptomycin, doxycycline	Death

Efe *et al*., 2009 [17]	1	Dyspnea	ST-depression T-wave inversion	Diffuse hypokinesia and ventricular dilatation (10-15% ejection fraction)	1:5120	Positive	No	Yes	Streptomycin (3 weeks, doxycycline (6 weeks	Cure

Khorvash *et al*., 2007 [20]	1	Fever, constitutional symptoms	Sinus bradycardia High T-waves	Septal hypokinesia, 30% ejection fraction	1:80	Positive	No	Yes	Doxycycline (6 weeks, rifampicin (6 weeks	Relapse

Hatipoglu *et al*., 2004 [14]	2	Dyspnea, arthritis, constitutional symptoms	NA	Pericardial effusion	1:320	Positive	Yes	No	Doxycycline (6 months), ofloxacin (6 months), rifampicin (6 months)	Cure
	
		Fever, dyspnea, retrosternal pain	NA	Pericardial effusion	1:640	Negative	Yes	No	Gentamicin (2 weeks, doxycycline (6 weeks, ciprofloxacin (6 weeks	Cure

Karagiannis *et al*., 2003 [22]	1	Fever, dyspnea	Atrial fibrillation	Large pericardial effusion	1:1280	Negative^b^	Yes	No	Streptomycin (2 weeks, doxycycline (6 weeks, indomethacin (2 weeks	Cure

Colmenero *et al*., 1996 [2]	2^c^	NA	NA	NA	NA	NA	Yes	Yes	Na	NA

Jubber *et al*., 1990 [23]	1^d^	Fever, dyspnea, constitutional symptoms	T-wave inversion	Moderate hypokinesia of interventricular septum and posterior wall of left ventricle	Negative	NA	No	Yes	Vibramycin, rifampicin	Cure

Rivera *et al*., 1988 [24]	1	Fever, chest pain, pericardial friction rub	NA	Moderate pericardial effusion	1:640	Positive	Yes	No	Rifampicin, doxycycline	Cure

Lulu *et al*., 1988 [19]	1^e^	NA	NA	NA	NA	NA	No	Yes	NA	NA

Ugartemendia *et al*., 1985 [12]	2	Fever, chest pain	NA	Severe pericardial effusion in both (tamponade)	1:320, 1:640	Positive	Yes	No	Cotrimoxazole, streptomysin, oxytetracycline	1 death, 1 cure

Gur *et al*., 1984 [25]	1	High remittent fever	Negative, biphasic or flat T-waves	NA	1:6400	Positive	No	Yes	Streptomycin (2 weeks, tetracycline (4.5 weeks	Relapse

^a^Constitutional symptoms include sweating, malaise, anorexia, weight loss, arthralgias, myalgias.

^b^This patient was already receiving antibiotic treatment.

^c^Two out of a series of 530 patients (one patient had pericarditis and one myocarditis).

^d^Diagnosis based on high titers of antibodies by ELISA.

^e^One out of 400 cases of brucellosis.

NA = not available.

In our first patient, the general symptoms were predominant and, contrary to other reports [12,14,24], the pericardial effusion was asymptomatic. This suggests that this unusual feature could be underestimated if echocardiography is not in routine use. However, both patients showed evidence of cardiac involvement on routine tests (chest radiography in the first and ECG in the second case) making the value of routine use of echocardiography in suspected cases of brucellosis questionable.

The cardiac damage may be due to a direct effect of the microorganism as suggested by pericardial fluid cultures [12], or by local deposit of immunocomplexes as seen in cardiac biopsies. In this context, the role of delayed diagnosis, as in our first patient, in the development of cardiovascular complications of brucellosis may deserve further evaluation. The diagnosis is based on the demographic and epidemiologic characteristics of the disease, the presence of symptoms, results of serological tests, isolation of the microorganism by blood or bone marrow cultures, and exclusion of other possible causes of pericardial effusion and myocarditis. Pericardiocentesis and heart biopsy remain the techniques of choice for supporting a definite diagnosis of *Brucella*-related involvement of the heart, but these interventional investigations are not easily performed for diagnosis in routine clinical practice and add no additional information when a safe diagnosis has already been made by other means.

We treated both our patients with triple antibiotic therapy only (without non-steroidal anti-inflammatory drugs or prednisolone). The response was complete in both, with no signs of relapses during the follow-up period. Although endocarditis was not found, both patients received treatment for three months because we believe that cardiac involvement (even in the absence of endocarditis) along with repetitive positive blood cultures cannot be safely considered an uncomplicated form of the disease while we lack evidence-based data from large prospective studies on the treatment of cardiovascular features of this disease in the absence of concomitant endocarditis.

## Conclusion

These cases further emphasize that *Brucella *can affect every organ and system of the human body [1,8,9,27,28]. The prevalence of involvement of the pericardium or myocardium in the absence of concomitant endocarditis in brucellosis may be underestimated because thorough echocardiographic studies in patients with brucellosis are lacking. However, both our patients showed evidence of cardiac involvement on routine tests and therefore, use of these tests seems to be essential in the assessment of febrile patients, especially where brucellosis is clinically and epidemiologically suspected. Echocardiography can be then used if such abnormalities are detected on routine tests. Nevertheless, we believe that in areas where the disease is endemic, it is reasonable to include brucellosis in the differential diagnosis of disorders that affect the pericardium or myocardium even in the absence of concomitant endocarditis, although large prospective echocardiographic studies in established brucellosis cases are needed in an attempt to definitively address the precise prevalence of cardiac complications of brucellosis. We are currently undertaking such studies.

## Competing interests

The authors declare that they have no competing interests.

## Consent

Written informed consent was obtained from the patients for publication of these case reports. A copy of the written consent is available for review by the Editor-in-Chief of this journal.

## Authors' contributions

GND and NKG had the original idea, and along with KPM wrote the first draft of the paper. GND, KPM and NKG investigated, treated and monitored the first patient, and IG and KK investigated, treated and monitored the second patient. NKG, AS and KPM collected all data from both patients and performed the literature search. IG and KK contributed to the final version of the paper, and GND, AS and NKG were major contributors to the final revised version of the manuscript. All authors read and approved the final manuscript.
